# Cost benefit analysis of malaria rapid diagnostic test: the perspective of Nigerian community pharmacists

**DOI:** 10.1186/s12936-016-1648-0

**Published:** 2017-01-03

**Authors:** Ifeoma Jovita Ezennia, Sunday Odunke Nduka, Obinna Ikechukwu Ekwunife

**Affiliations:** 1 Department of Pharmacy, Enugu State University Teaching Hospital, Enugu, Nigeria; 2 Department of Clinical Pharmacy and Pharmacy Management, Nnamdi Azikiwe University, Awka, Nigeria; 3 Collaborative Research Group for Evidence-Based Public Health, Department of Prevention and Evaluation, Leibniz Institute for Prevention Research and Epidemiology – BIPS / University of Bremen, Bremen, Germany

**Keywords:** Malaria, Rapid diagnostic test, Cost-benefit analysis, Willingness-to-pay, Contingent valuation, Community pharmacy, Nigeria

## Abstract

**Background:**

In 2010, the World Health Organization issued a guideline that calls for a shift from presumptive to test-based treatment. However, test-based treatment is still unpopular in community pharmacies. This could be due to unwillingness of customers to spend extra finance on rapid diagnostic test (RDT). It could also result from lack of interest from community pharmacists since they may perceive no financial gain attached to this service. This study assessed the cost-benefit of test-based malaria treatment to community pharmacists.

**Methods:**

The study was a community pharmacy-based cross sectional survey. Potential benefit of RDT was determined using customers’ willingness-to-pay (WTP) for service. Average WTP was estimated using contingent valuation. Binary logistic regression was used to assess correlates of WTP acceptance while multiple linear regression was used to model the relationship between the independent variables and WTP amount. Cost associated with provision of RDT was estimated from provider’s perspective. Probabilistic sensitivity analysis was used to capture parameter uncertainty. Benefit-cost ratio (BCR) was calculated to determine study objective.

**Results:**

A total of 135 out of 235 participants (57.4%) responded to the WTP question. Of this subset, 111 participants (82.2%) preferred RDT before malaria treatment. Average WTP [minimum–maximum] was US$1.23 [US$0.0–US$5.03]. Educated participants had 1.8 times higher odds of WTP for RDT. Participants that understood RDT as described in the questionnaire had 18.3 times higher odds of WTP for RDT compared to participants that did not understand RDT as described in the questionnaire. Additionally, a unit increase in level of education (e.g. from primary to secondary school) led to US$0.298 increase in WTP amount for RDT. Also, a unit increase in malaria frequency (e.g. from ‘never’ to ‘rarely’) led to US$0.293 decrease in WTP amount for RDT. Average cost [minimum–maximum] of RDT test kit and pharmacist time spent in administering the test were US$0.15 [US$0.13–US$0.17] and US$0.41 [US$0.18–US$0.52], respectively. BCR of test-based malaria treatment was 6.7 (95% CI 6.4–7.0).

**Conclusion:**

Test-based malaria treatment is cost-beneficial for pharmacy practitioners. This finding could be used as an advocacy tool to increase community pharmacists’ interest and uptake of test-based malaria treatment.

**Electronic supplementary material:**

The online version of this article (doi:10.1186/s12936-016-1648-0) contains supplementary material, which is available to authorized users.

## Background

Malaria is a major public health challenge and a leading cause of morbidity and mortality in Nigeria. The disease causes a substantial health burden. Nigeria and the Democratic Republic of Congo contribute more than 35% of the global total of estimated malaria deaths [1]. Malaria accounts for about 60% of outpatient visits, 30% of hospitalization and it is believed to contribute up to 11% of maternal mortality, 25% of infant mortality, and 30% of under-5 mortality [2]. The disease is estimated to retard Nigeria’s gross domestic product (GDP) by 40% annually and costing approximately 480 billion naira (approximately US$1.5 billion) in out-of-pocket treatments, prevention costs, and loss of man hours [2].

Approaches in malaria diagnosis include clinical diagnosis, microscopic diagnosis, molecular diagnosis and serology [3], with clinical and presumptive diagnosis being the conventional diagnostic method [4]. However, clinical diagnosis using fever as an indicator has been shown to be a sensitive indicator of clinical malaria in children <5 years, but not in older children and adults [5]. Other diagnostic approaches require trained staff, expensive and fragile equipment, and electricity supply among others. These requirements and inherent potential for human and technical errors have been shown to result in misdiagnosis and over-diagnosis of malaria [3, 4, 6–9]. This over-diagnosis of malaria exposes patients to needless anti-malarial therapy, waste of resources in resource-scarce setting, and may likely contribute to the development of drug resistance [10, 11]. The problem of misdiagnosis and over treatment of malaria led to the development of a more reliable, field-suitable and cost-effective diagnostic tool–rapid diagnostic test (RDT) [12, 13]. Rapid diagnostic test, based on the detection of Plasmodium antigens in a little drop of the patients’ blood, saves cost and time while giving an almost instant result. It generally has high level of sensitivity and specificity, although its accuracy varies between brands, location and epidemiological setting [14, 15]. The accuracy and reliability of RDT made it a staple in malaria control programmes and a first choice malaria diagnostic tool [16, 17]. RDTs testing for falciparum malaria were shown in a Cochrane review to be very specific (range of about 92–100%) meaning that only 0–8% of patients who test positive would not actually have the disease [18]. Furthermore, they were shown to be very sensitive (range of about 91–99%) meaning that only 1–9% of people with falciparum malaria would actually get a negative test result [18].

To effectively diagnose and treat malaria, World Health Organization in 2010 issued guideline that moved from select implementation of malaria testing to adoption of universal testing including children under 5 years [19]. Practically, this implies that in all suspected cases, the diagnosis of uncomplicated malaria should be confirmed using a parasitological test (either microscopy or RDT) prior to treatment [20, 21]. Arguments in favour of the shift to test-based management of malaria were of the opinion that the factors which justified presumptive approach were no longer valid namely: high malaria transmission; availability of affordable yet effective antimalarial drugs; and lack of appropriate diagnostic tools. They also argued that test-based approach would lead to improvement in the management of non-malaria febrile illness [18, 22–24]. Consistent with the WHO recommendation, Nigeria in 2011 updated the national malaria treatment guideline to reflect universal testing before treatment for suspected cases of malaria [25].

In most African countries, large proportions of the population utilize the private sector, particularly drug shops, as their first point of care for fever and malaria treatment [26, 27]. This is similar to the situation in Nigeria, in which nearly 60% of Nigerians seek treatment for malaria at drug shop outlets in the private health sector, composed of licensed community pharmacies (or referred to as pharmacies) and loosely regulated proprietary and patent medicine vendors (PPMV) [15, 28, 29]. This treatment-seeking behaviour is attributed to patients’ convenience, availability of familiar drugs, affordability, reduced waiting hour and proximity [30].

The expected responsibilities of community pharmacists in the prevention and management of malaria is anchored on the pharmaceutical care principle of responsible provision of drug therapy in order to improve patients’ quality of life [31, 32]. With anti-malarial drugs being an ‘over-the-counter’ medication in Nigeria, community pharmacists have the responsibility of ensuring that patients seeking anti-malarial drugs truly have malaria before recommending the appropriate anti-malarial drugs in line with WHO call for routine diagnostic test in all suspected cases of malaria prior to treatment. Despite the call for a shift from presumptive to test-based treatment, uptake of RDT by formal and informal drug vendors remain low, as malaria treatment is still mostly based on presumptive approach [33, 34].

The non-provision of RDT service in Nigerian community pharmacies could be connected to the fact that patients with suspected cases of malaria may not be willing to spend extra money for RDT in addition to anti-malarial drugs. Community pharmacists may also be interested in making more sales for better financial gains rather than spending time to offer such RDT service. Thus, community pharmacists may need to be incentivized to offer RDT service. A previous Nigeria-based study assessed the willingness to pay (WTP) for RDT in health centres and households ex ante and ex post to malaria diagnosis [35]. However, review of literature showed that no study has assessed the financial benefit of test-based malaria treatment from the community pharmacist’s perspective. This study was, therefore, aimed at assessing the willingness to pay (WTP) for malaria RDT among patients visiting community pharmacies for malaria treatment using a contingent valuation method and the cost-benefit of test-based malaria treatment to the community pharmacy practitioner.

## Methods

### Study design and study population

**Fig. 1:**
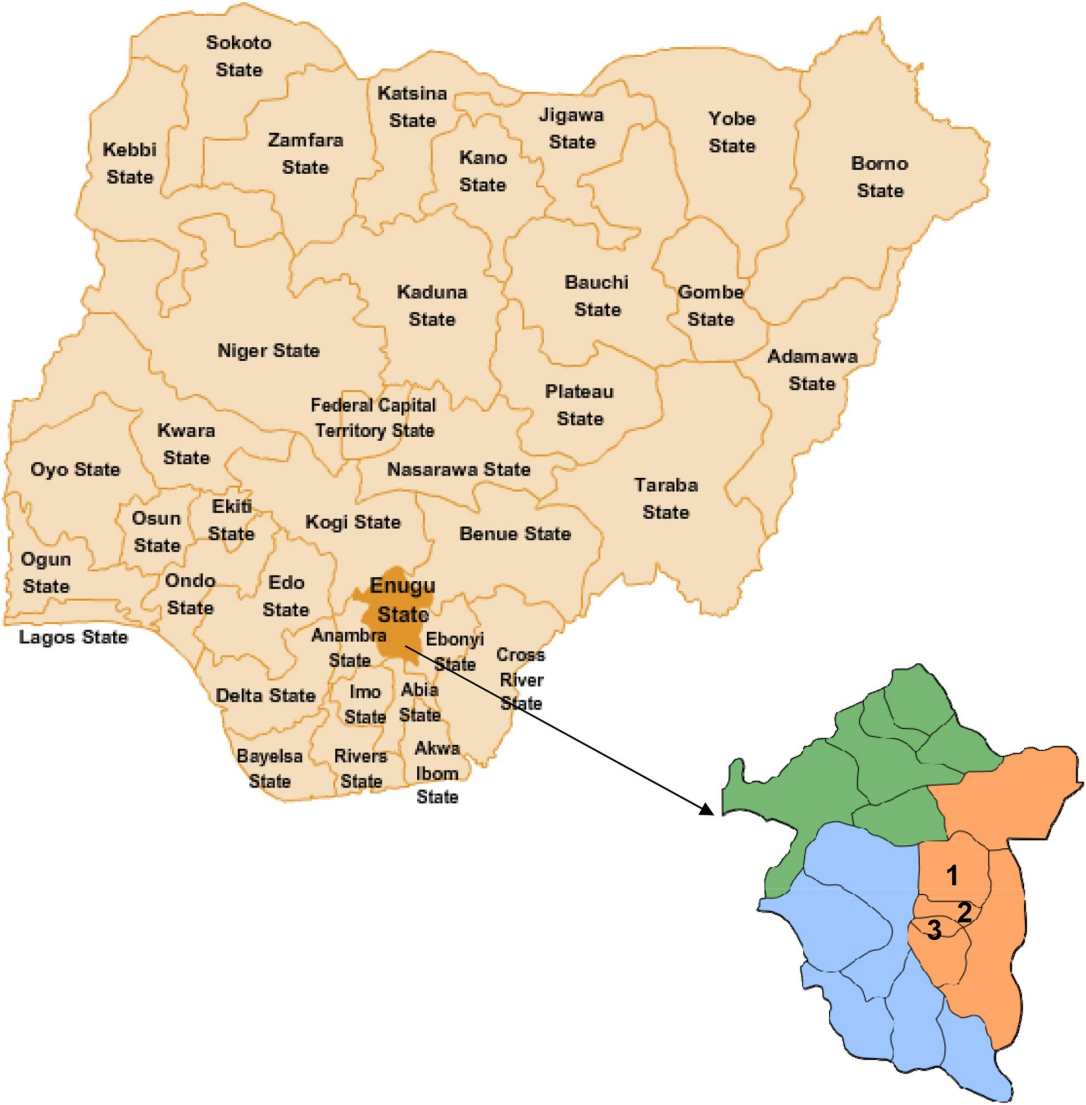
Map of Nigeria highlighting the local government areas (LGA) were the study was conducted in Enugu State. *1* = *Enugu North LGA; 2* = *Enugu South LGA; 3* = *Enugu West LGA*

As shown in Fig. 1, the pharmacy-based cross sectional study was carried out in Enugu metropolis, in south eastern part of Nigeria, from November 2015 to March 2016. Enugu state, also known as the Coal City State, is located within latitude 6°.00′N and 7°.00′N and longitude 7°.00′E and 7°.45′E, and has Ibos as its indigenous ethnic group [36]. Enugu has an estimated population of 3,257,298 [37], and there were 98 community pharmacies registered with Pharmacist Council of Nigeria in the state as at January 2015. Enugu was purposively selected since different cadres of pharmacies operate in the city.

Community pharmacies selected for the study were those registered with Pharmacist Council of Nigeria (PCN) and those that have a full or part time pharmacist(s) working in the pharmacy. With a population of 98 pharmacies and assuming a confidence level of 95% with a confidence interval of ±12, a sample size of 40 pharmacies was estimated to be adequate for the survey [38]. Stratified sampling technique was employed to select pharmacies for the study ensuring the inclusion of persons from high, middle and low socioeconomic class. Enugu metropolis was divided into ten strata namely: Trans-ekulu, Abakpa, New heaven, Coal camp, Independence layout, Ogui, Achara layout, Uwani, GRA and Ogbete, from which four community pharmacies were randomly selected from each stratum. Patients not less than 18 years, who had the ability to read and write, those with suspected cases of malaria, and those that gave consent were invited to participate in the study.

### Benefit estimate: willingness-to-pay for RDT

Contingent valuation approach using the payment card technique was used to estimate the average maximum willingness-to-pay (WTP) among the survey participants. Self administered questionnaire was developed for the WTP assessment (Additional file 1). The questionnaire consisted of three sections. Section A contained socio-demographic information of the respondents. Section B consisted of attitude of respondents towards malaria. Section C assessed respondents willingness to pay (WTP) after a brief scenario description. RDT preference was assessed based on the response to the following question: “Will you be willing to pay for RDT so as to be tested for malaria in the pharmacy before initiation of treatment”? The follow-up question was used to assess willingness to pay (WTP) of those that prefer RDT before treatment. The question reads as follows: “How much will you be willing to pay for the RDT from the scale below. Offered WTP values in the payment card ranged from 50 Naira to more than 1000 Naira (equivalent to US$0.25–US$5). The maximum amount they were willing to pay was considered as their perceived monetary benefit of the RDT. This is in accordance with welfare economic theory which states that the benefit to an individual of a service or intervention is defined as the individual’s maximum willingness to pay for the service or intervention.

The elicitation format employed in determining respondents’ WTP was the payment scale/card and open-ended question. The payment scale presented respondents with a range of values to choose from, in a vertical list from the lowest bid (top) to the highest bid (bottom) in increments. The open-ended question on the other hand was employed if the maximum WTP was greater than the highest bid in the scale and also to find out respondent’s reason for choosing their maximum WTP amount. Different payment scales with different ranges were randomly allocated across the study sample to avoid range bias. The questionnaire was face validated, to assess the presentation as well as the relevance of the questionnaire, and pilot tested to assess feasibility. Modifications were made to the questionnaire based on any identified problem(s) during the pilot study. The final questionnaire was distributed in the selected pharmacies in Enugu after obtaining oral informed consent from respondents.

Responses to the WTP question were grouped into two categories: those that prefer RDT before treatment and those that prefer presumptive treatment (RDT rejecters). The response to WTP question served as the dependent variables in a multivariate binary logistic regression. The independent variables were re-categorized into the following variables:

i. Socio-economic data: sex, age of respondents (4 dummy codes for 21–30 years, 31–40 years, 41–50 years, and >50 years), number of children (4 dummy codes for 2, 3, 4, and >4 children), level of education (4 dummy codes for primary, secondary, tertiary and post-tertiary education), average household income (5 dummy codes for US$50–US$251, US$251–US$502, US$502–US$1256, US$1256–US$2512, >US$2512), whether respondent is employed (5 dummy codes for farming, civil servant, trader, self-employed, others).
ii. Malaria and RDT experience—Frequency of malaria in household (4 dummy codes for rarely, sometimes, often, always); importance of testing, and understanding of RDT.

Additionally, linear regression was used to model the relationship between the independent variables and the amount the respondents were willing-to-pay for RDT. Data was initially coded and transferred to Microsoft Excel (Microsoft Office 2010). Further re-categorization of data (i.e. creation of dummy variables) was done in Microsoft Excel before it was imported to SPSS (Version 20). Logistic regression and linear regression were performed employing the backward conditional method and the independent variables above serving as the predictor variable. A two-tailed significance value of 0.05 was used.

### Cost estimate

Cost was estimated from the health provider’s perspective. The prevailing community pharmacists’ salaries were used. The salaries of the three basic cadres of pharmacists that work in community pharmacies were obtained from the study sites. Since the three basic cadres of pharmacists earn differently, they were used to obtain the minimum, most likely and maximum values of staff cost. Using the average working hour, the cost of the 15 min of pharmacists’ time was deduced to represent the time spent on administration of malaria RDT service [35, 39, 40]. The minimum, average and maximum distributor’s price for malaria RDT kit was obtained for all the commonly available RDT kits in Enugu. Cost of consumables such as alcohol for disinfection, lancets, and hand gloves were not included since they are covered in the unit cost of RDT kit (i.e. they were already contained in the RDT kit).

### Benefit-cost analysis

Cost benefit of test-based malaria treatment with RDT from the community pharmacists’ perspective was calculated by dividing benefits over costs, referred to as a benefit-cost ratio (BCR). A ratio >1 demonstrated a positive return on investment. In this analysis the potential benefit was measured as WTP for RDT service. The costs included the pharmacist’s time for administering RDT and the cost of RDT kit.

Using the average estimates of parameters to calculate BCR will be misleading since the cost parameters varied widely in reality. Therefore, probabilistic sensitivity analysis (PSA) approach was used to account for parameter uncertainty [41]. The PSA allowed exploration of the joint uncertainty in costs and benefit estimates. Triangular distributions of each parameter were used for PSA calculation. A thousand Monte Carlo simulations were run and the mean [95% confidence interval] of BCR was presented. Specifically, a point estimate was drawn randomly from the distribution of each parameter used in estimating BCR. This was repeated for 1000 times (a thousand iterations) and then the average of the 1000 iterations with 95% confidence interval calculated. Monte Carlo simulation was conducted using Microsoft Excel 2010.

## Results

### Characteristics of respondents

A total of 235 respondents successfully answered the questionnaire, one half were male while the other half were female. Majority of the respondents who participated in the survey were aged 21–30 years, single and from Ibo tribe. Most of the respondents had a formal education, with more than half (53.7%) with tertiary education. About half of the respondents (57.5%) reported earning a monthly income of US$50–US$502 (Table 1).

**Table 1:** Socio-demographic characteristics of respondents (n = 235)

Characteristics	Frequency (%)
*Sex*	
Male	113 (49.1)
Female	117 (50.9)
*Age (years)*	
18–20	22 (9.6)
21–30	114 (49.6)
31–40	60 (26.1)
41–50	19 (8.3)
>50	15 (6.5)
*Tribe*	
Hausa	13 (5.6)
Igbo	200 (86.6)
Yoruba	10 (4.3)
Others	8.5 (3.5)
*Marital status*	
Single	139 (59.7)
Co-habiting	7 (3.0)
Married	85 (36.5)
Divorced	1 (0.4)
Widowed	1 (0.4)
*No of children*	
One	15 (17.4)
Two	24 (27.9)
Three	15 (17.4)
Four	15 (17.4)
≥Five	17 (19.8)
*Level of education*	
No education	4 (1.8)
Primary	7 (3.1)
Secondary	38 (16.7)
Tertiary	122 (53.7)
Post tertiary	56 (24.7)
*Occupation*	
No job	49 (22.1)
Farming	10 (4.5)
Civil servant	48 (21.6)
Trader	16 (7.2)
Self employed	57 (25.7)
Others	42 (18.9)
*Monthly income*	
<US$50 (N10,000)	33 (18.4)
US$50–US$251 (₦10,000–₦50,000)	67 (37.4)
US$251–US$502 (₦50,000–₦100,000)	36 (20.1)
US$251–US$502 (₦50,000–₦100,000)	29 (16.2)
US$1256–US$2512 (₦250,000–₦500,000)	9 (5.0)
>US$2512 (>₦500,000)	5 (2.8)

### Average WTP value for RDT and its predictors

**Fig. 2:**
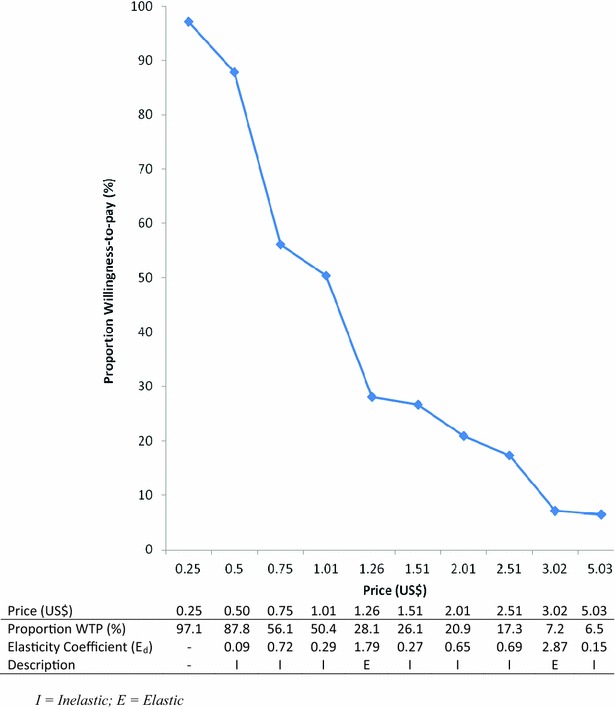
Willingness-to-pay demand curve and price elasticity of demand

Table 2 shows the WTP value for malaria RDT kit. A total of 135 out of 235 participants (57.4%) responded to the WTP question. Of this subset, 111 participants (82.2%) stated that they prefer RDT before malaria treatment while 24 (17.8%) stated that they prefer presumptive treatment. The reason given by majority of the respondents for rejecting RDT and choosing presumptive treatment was majorly due to financial considerations. In other words, patients who were not willing to pay for RDTs may still prefer to get tested before treatment if the testing were free. The stated average WTP [minimum–maximum] by the respondents was US$1.23 (US$0.0–US$5.03). Fifty percent of the respondents stated US$1.01 as their WTP amount while the most frequently stated WTP amount was US$0.50. Figure 2 shows the willingness to pay demand curve which depicts the relationship between the price of RDT and the percentage of patients willing to pay to get tested. The demand curve had a negative slope, moved downward from left to right, consistent with the law of demand. This implied that as the price of RDT increased, the percentage of patients willing to pay decreased, all things being equal.

**Table 2:** WTP amount for malaria rapid diagnostic test and its predictors (n = 135)

Statistics	WTP per test (US$)
Mean	1.23
Median	1.01
Mode	0.50
Percentiles	
20	0.50
90	2.51

	Dependent: RDT acceptance (=1), n = 111 and RDT rejection (=0), n = 24		
	B	b(exp)	Sig
*Binary logistic regression*			
Education	0.608	1.836	0.123
Understanding of RDT	2.909	18.343	0.003
Constant	−3.989	0.019	0.003
Nagelkerke R^2^	0.181		

	Dependent: Maximum WTP amount, n = 111		
	B	Std. Error	Sig
*Multiple linear regression*			
Education	0.298	0.130	0.024
Malaria frequency	−0.293	0.120	0.017
Constant	0.836	0.436	0.058
R^2^	0.096

Logistic regression examining predictors of RDT acceptance showed that educated participants had 1.8 times higher odds of WTP for RDT before initiation of malarial treatment (Table 2). Participants that understood RDT from the description provided in the questionnaire had 18.3 times higher odds of preferring to undergo RDT before the initiation of malarial treatment compared to participants that did not understand RDT from the description provided in the questionnaire. The predictive capacity of the model was 18.1%. Additionally, multiple linear regression examining predictors of WTP amount for RDT showed that two variables (education and frequency of malaria infection) predicted WTP amount for RDT, F(2, 97) = 5.169, p = 0.007, R^2^ = 0.096 (Table 2). Each of the two variables added statistically significantly to the prediction, p < 0.05. Specifically, a unit increase in level of education (e.g. from primary to secondary school) led to US$0.298 increase in WTP amount for RDT. Also, a unit increase in malaria frequency (e.g. from ‘never’ to ‘rarely’) led to US$0.293 decrease in WTP amount for RDT.

### RDT cost and cost-benefit analysis

Three categories of community pharmacists (community pharmacy owners, superintendent community pharmacists and locum community pharmacists) were interviewed to obtain average (minimum–maximum) community pharmacist’s salary. Pharmacist’s time spent in administering RDT was prorated from the average community pharmacist’s salary. Fifteen minutes was assumed to be pharmacist’s time spent on administering RDT. This was based on observation of a trial RDT procedure in a community pharmacy as well as reports from literature [35, 39, 40]. Cost of RDT kit was obtained from major distributors of pharmaceuticals and medical products in Ogbete main market, Enugu. The average cost (minimum–maximum) of 15 min of pharmacist time spent in administering the test and RDT kit were US$0.41 (US$0.18–US$0.52) and US$0.15 (US$0.13–US$0.17), respectively. The BCR of the test-based malaria treatment was 6.7 (95% CI 6.4–7.0). Further details are shown in Table 3.

**Table 3:** Cost and benefit data

Variable	Average [min–max] (US$)	Distribution	Source
Staff salary/month			
Locum (n = 36)	67.56 [60.30–100.50]		Interview
Full time pharmacist (n = 22)	389.45 [251.26–452.26]		Interview
Superintendent pharmacist (n = 27)	402.01 [351.76–502.51]		Interview
Staff cost (15 min)	0.41 [0.18–0.52]	Triangular	–
RDT kit (n = 12)	0.15 [0.13–0.17]	Triangular	Market survey
WTP for RDT	1.23 [0.0–5.03]	Triangular	Survey

## Discussion

This study assessed the willingness to pay (WTP) for malaria rapid diagnostic testing among patients visiting community pharmacies for anti-malarial drugs. The aim of the study was to evaluate the cost-benefit of offering test-based malaria treatment from the community pharmacist’s perspective. The findings showed that average WTP [minimum–maximum] for RDT was US$1.23 [US$0.0–US$5.03] and return on invested time was approximately 7 times for the community pharmacist. Educated patients and hose with good comprehension of RDT from the description provided in the questionnaire were more likely to accept testing with RDT before treatment. Similarly, being educated was related with higher value placed on RDT while patients with higher frequency of malaria infection placed lower value on RDT.

The results obtained in this study were similar to previously published studies. This study showed that the majority of the participants were willing to pay an average of US$1.23. This is comparable to the findings of Uzochukwu et al. [35] which showed a mean ex-ante willingness to pay for RDT of US$1.18 in the urban area. Also, the finding that educated participants were more likely to prefer test-based malaria treatment is similar to a study in Uganda, which showed that a higher WTP was associated with respondents who had higher level of education [42].

This study has practical inferences for maximizing the utilization of test-based malaria treatment in community pharmacies and proprietary and patent medicine vendors (PPMV), particularly for malaria endemic regions. Among the participants that responded to the WTP question, a total of 82.2% of the respondents were willing to pay for RDT services, indicating a high demand for the service. The most frequently stated willingness to pay amount is greater than the existing market price of RDT, reflecting that majority of the participants have value for RDT and are willing to utilize it if made available. Notwithstanding, a number of factors may have influenced those that rejected RDT or those that did not answer the WTP question. This may include difficulty in placing value on an unfamiliar commodity and viewing of RDT as an extra cost in addition to the cost of medication, thus perceiving it as unaffordable [42]. This highlights an immense need for awareness and education of the masses as well as pharmacy practitioners on the importance and benefits of test-based malaria treatment in order to increase its utilization. Such intervention could lead to overall change in public perception of RDT [33]. It may also be important to explore offering RDT and malaria treatment as a single bundled commodity rather than two separate commodities [42]. Such strategy could improve uptake of RDT. Finally, it is important to explore the use of subsidies for RDT especially as financial constraint was a major reason for those that choose presumptive treatment rather that test-based treatment [35, 42].

Furthermore, findings from our study showed a BCR of 6.7. This implies that test-based malaria treatment will generate a return that amounts to approximately 7 times the cost of investment. Thus, the positive return on investment could be used as an advocacy tool to increase the interest of community pharmacy practitioners on test-based malaria treatment. The pharmacists were interviewed to ascertain why they do not make RDTs available in their pharmacies. Although not originally part of the study objectives, interview of the pharmacists revealed that most of them perceive the results of RDT as inaccurate especially when the results are negative. Majority of the pharmacists interviewed believe that a patient presenting with signs and symptoms of malaria must test positive to RDT. Similarly, some of the pharmacists view RDT as an additional cost to the cost of malaria medication and as such, they believe that patients may not be willing-to-pay for RDT. Lastly, most pharmacists think test-based treatment will reduce their sales especially as they deem their practice a profit-oriented venture.

There is a caveat to promoting RDTs in community pharmacies which is pertinent to mention. There is a need to regulate the price of RDT in the community pharmacies so as to avoid outrageous mark up. For instance, Poyer et al. [43] showed that median RDT prices were higher in pharmacies compared to other health care centers. Uncontrolled RDT price in community pharmacies could contribute substantial cost to the treatment of malaria with the end result felt mostly by those in the lower socio-economic strata. Such will cause treatment of malaria to be catastrophic especially considering that about 99 million Nigerians (58%) live with less than US$1.25 per day [44]. Additionally, RDT accuracy in malaria case management is highly user-dependent despite their apparent simplicity [45]. Many of the errors with malaria RDT kit are related to the post-analytical phase, i.e. wrong reading of the test and control lines and incorrect interpretation of the results [45, 46]. Therefore, quality assessment on the use of malaria RDT test in pharmacies should be promoted through monitoring. For instance, Pharmacist Council of Nigeria (the body in-charge of regulating pharmacy practice in Nigeria) or Association of Community Pharmacists of Nigeria (ACPN) could incorporate monitoring of RDT use in pharmacy premises as part of their responsibilities.

A drawback to the findings is the issue of generalizing the result which should be considered while interpreting the result. Notwithstanding, effort was made to select a city with a fair representation of community pharmacy practice in Nigeria especially since all cadres of community pharmacies operate in Enugu. Secondly, community pharmacies in Nigeria tend to cluster around the cities and this means that people living in rural areas are potentially excluded from the study. This is evident as about 78% of the study participants had tertiary education which contrast markedly with the national average of 7.1% [47]. Also, the WTP value obtained in this study should be considered in view of bias associated with payment scale/card elicitation format. Participants could have been influenced by the range of values chosen for the payment scale question design instead of their true maximum WTP values. However, in order to mitigate this bias, several payment scales with different ranges were used. Since only patients with suspected cases of malaria infection were included, their possible illness could have made them more likely to pay for RDT even under circumstances where they would have behaved otherwise. Lastly, the small sample size of RDT rejecters could have induced a systematic bias as logistic regression could overestimate odd ratios in studies with small to moderate sample size [48].

## Conclusion

Malaria testing using RDT before recommending malaria treatment for those that tested positive is a cost-beneficial practice for community pharmacy practitioners with a return on investment of 6.7 times. This finding could serve as an advocacy tool to increase community pharmacy practitioners’ interest and uptake of test-based malaria treatment. Consequently, increase in uptake of test-based malaria treatment would help to reduce over-diagnosis of malaria, over-prescription of anti-malarial drugs, and invariably the cost of treatment especially if the testing is done appropriately and community pharmacists and patients adhere to the results.

## Additional file

**Additional file 1.** Willingness-to-pay questionnaire
