# Supplementary material for: Cost benefit analysis of malaria rapid diagnostic test: the perspective of Nigerian community pharmacists
Source: Malar J. 2017 Jan 3;16:7. doi: 10.1186/s12936-016-1648-0 (PMC5210296; doi:10.1186/s12936-016-1648-0)
Supplement: Supplementary file 1 — Additional file 1. Willingness-to-pay questionnaire [file 12936_2016_1648_MOESM1_ESM.doc]

**Willingness to Pay for malaria Rapid Diagnostic Test Questionnaire**

**Introduction**

*This study is designed to find out the price which an individual would be willing and can afford to pay for the use of malaria rapid diagnostic test (RDT) in the diagnosis and treatment of malaria in community pharmacies. Findings from this study will help us (community pharmacists) to serve you better. It would be useful to obtain some information about you bearing in mind that this questionnaire is anonymous. Hence, I plead for your sincere opinion about the questions below as there is no right or wrong answers. Thank you for your time.*

*Ezennia Ifeoma*

**Section A: Demographic Information**

Please tick the appropriate box.

1. What is your sex? Male [ ] Female [ ]
2. How old are you? 18- 20 [ ] 21- 30 [ ] 31- 40 [ ] 41- 50 [ ] above 50 [ ]
3. Which tribe are you from? Hausa [ ] Ibo [ ] Yoruba [ ] Any other (specify) __________
4. What is your marital status? Single [ ] Married [ ] Divorced [ ] Widowed [ ]
5. How many children do you have? 1 [ ] 2 [ ] 3 [ ] 4 [ ] Above 4 (specify) ____________
6. What is your level of education? No education [ ] Primary [ ] Secondary [ ] Tertiary [ ] Post tertiary [ ]
7. What is your occupation? No job [ ] Farming [ ] Civil servant [ ] Trader [ ]

Self employed [ ] others (please specify) _______________________________________

1. What is your income per month in Naira? Below 10,000 [ ] 10,000 – 50,000 [ ] 50,000 – 100,000 [ ] 100,000 – 250,000 [ ] 250,000 – 500,000 [ ] Above 500,000 [ ]

**Section B: Fact Sheet**

*Malaria is caused by the presence of malaria parasites in the blood. It is usually diagnosed by examining the blood for the presence of parasites using a microscope which is normally carried out in the laboratories. Recently, a newer and more simplified way of carrying out this test through the use of a* ***Rapid diagnostic test (RDT)*** *was introduced.*

*RDT requires only a drop of blood through a finger prick. This makes it easy and safe to use with minimal pain or discomfort and it takes about 15 minutes to obtain the results. The test is usually performed by a trained health worker in a health setting.*

*The benefit of carrying out malaria test prior to treatment is to ascertain the presence of malaria parasite, hence, avoiding unnecessary drug therapy. Consequently, it reduces costs of treatment since only people that tested positive to the parasite will receive anti-malarial drugs. The payment for the rapid diagnostic tests (RDTs) will be paid out of the individuals pocket before carrying out the test.*

**Questions**

1. Did you understand what an RDT is from the description above? Yes [ ] No [ ]
2. RDTs are devices which can be used to test if a person has malaria parasite? True [ ] False [ ]
3. How often do you or any member of your household suffer from malaria? Always [ ] Very Often [ ] Sometimes [ ] Rarely [ ] Never [ ]
4. Where do you seek for medical help in cases of suspected malaria? Hospital [ ] Pharmacy [ ] Laboratory [ ] Chemist/Patent drug dealers [ ] others__________________ *(Tick all that apply)*
5. How often were you tested for the presence of the parasite before treatment? Always [ ] Very Often [ ] Sometimes [ ] Rarely [ ] Never [ ]
6. Have you or any of your household had malaria in the past 2 weeks? Yes [ ] No [ ]
7. Were you tested and diagnosed of malaria before your present visit to the pharmacy for malaria treatment? Yes [ ] No [ ]
8. Do you think that it is important to ascertain the presence of malaria parasite before treatment? True [ ] False [ ]
9. Have you heard of RDT before now? Yes [ ] No [ ]
10. If yes, where did you first hear of RDT? Hospital [ ] Pharmacy [ ] A neighbor [ ] Radio/Television [ ] Books [ ] School [ ] Others (specify)________________________
11. Has the RDT test kit being used on you before? Yes [ ] No [ ]
12. If yes, where was the test carried out? Hospital [ ] Pharmacy [ ] Laboratory [ ]
13. Will you be willing to pay for the RDT to be tested for malaria in the pharmacy before the initiation of treatment? Yes [ ] No [ ]

*NB: If ‘****Yes’*** *to question 13, please proceed to question 14. If ‘****No’*** *to question 13, please skip question 14 and proceed to question 15.*

1. How much will you be willing to pay for the RDT from the scale below

N0

N25

N50

N100

N150

N200

N250

N300

N350

N400

N500

N600

N800

N 1000

N ­­­_________

1. What was your reason for choosing the maximum amount (or no amount)?

________________________________________________________________________

________________________________________________________________________

*NB: Please for question 14, put a tick mark next to the amounts that you are sure you would pay, put a cross mark next to the amounts you are sure you would not pay, and circle the maximum amount you will be willing to pay.*
